# A Rare and Late Postoperative Complication of Nissen Fundoplication: Mixed Hiatus Hernia

**DOI:** 10.5005/jp-journals-10018-1285

**Published:** 2019-02-01

**Authors:** Murat Kekilli, Zeynal Dogan, Fatih Karaahmet

**Affiliations:** 1 Department of Gastroenterology, Ankara Training and Research Hospital, Ankara, Turkey; 2 Department of Gastroenterology, Ankara Training and Research Hospital, Ankara, Turkey; 3 Department of Gastroenterology, Ankara Training and Research Hospital, Ankara, Turkey

**Keywords:** Gastroesophageal reflux disease, Mixed hiatus hernia, Nissen fundoplication.

## Abstract

**How to cite this article:** Kekilli M, Dogan Z, Karaahmet F. A Rare and Late Postoperative Complication of Nissen Fundoplication: Mixed Hiatus Hernia. Euroasian J Hepatogastroenterol, 2018;8(2):172.

Nissen fundoplication (NF) is generally considered to be safe and effective for treatment of gastroesophageal reflux disease (GERD) and hiatal hernia. Complications of NF include dysphagia, diarrhea and flatulence, recurrent heartburn and atypical symptoms. The fundoplication can also come undone over time in about 5 to 10% of cases^1^. We want to present a patient with mixed (sliding and paraesophageal) a hiatal hernia developed after Nissen fundoplication surgery.

**Fig. 1:**
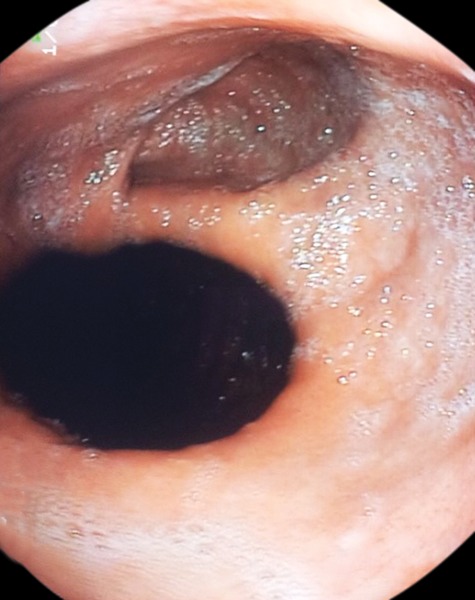
An upper gastrointestinal endoscopy shows mixed hiatus hernia

A 37-years old man with dyspeptic complaints was admitted to the gastroenterology outpatient clinic. In his history, 5 years ago, he had a surgical operation of Nissen fundoplication due to medically uncontrolled gastroesophageal reflux symptoms related to sliding hiatus hernia. Relief of symptoms had been provided after surgery for about three years. After then, he has had symptoms including heartburn, worsening regurgitation for two years. Physical examination and laboratory tests were normal. An upper gastrointestinal endoscopy demonstrated gastritis and the features of sliding and paraesophageal hiatus hernia (Fig. 1). Biopsies obtained from antrum, angulus, and corpus during endoscopy were negative for *Helicobacter pylori* infection. Esomeprazole 40 mg day and lifestyle chancing including weight loss, elevation of the head of the bed, and elimination of dietary triggers were suggested. Relief of symptoms were provided after four weeks of treatment. Minimal reflux symptoms are following a patient by three months intervals.

Several late postoperative complications of NF particularly rare occurrences. This operation can also come undone over time in about 5 to 10% of cases, leading to a return of symptoms.^2^ If these options do not relieve symptoms, surgery may be needed. Therefore, the success of Nissen fundoplication requires assistance between the gastroenterologist and surgeon to avoid undesirable conditions.
